# Beyond a literacy model for psychiatry in the mass media

**DOI:** 10.1192/bjb.2023.63

**Published:** 2024-08

**Authors:** Gavin Miller

**Affiliations:** 1 University of Glasgow, UK

**Keywords:** Public understanding of psychiatry, psychiatry in the mass media, celebrity, broadcasting, publishing

## Abstract

Professional statements suggest that psychiatrists engage in media work to supply a general audience with medical knowledge informed by relevant professional expertise. However, media work may be motivated by interests other than disinterested service to the well-being of the public, such as fame, money and a platform for one's wider views. The role of media psychiatrist is also crucially shaped by the unpredictable needs of a complex media ecology and marketplace. Furthermore, the properties of the media, and different forms within them, bring implicit meanings such as the wider authorisation of therapeutic self-reflection or the promotion of para-social intimacy. Finally, the media psychiatrist may function as an entrepreneur, converting the currency of celebrity into new forms of cultural, social and political capital. Professional guidelines for media work should be updated in light of such observations.

Psychiatrists work in the mass media, as do other mental health professionals. They write for newspapers and the general book trade; they are interviewed on radio and television; and they present and devise programmes.

How do the mental health professions, including psychiatry, understand media work? The Royal College of Psychiatrists’ *Public Education Handbook* states that media work is ‘the gateway to better public understanding, breaking down stigma and improving knowledge about mental health and illness’.^1^ The Handbook offers useful tips on various types of media work and encourages psychiatrists to correct stigmatising mass media representations. It also reminds psychiatrists that they should not offer professional opinions on individuals ‘whose behaviour – often criminal or violent – has caused public concern’ (an ethical principle borrowed from APA guidelines^2^). In the allied profession of psychology, British Psychological Society guidance on psychology and media productions advises commissioners and producers that psychology, glossed as ‘the science of human experience and behaviour’, ‘can have a major positive effect on society’ by ‘enhancing wellbeing through better public knowledge about psychological processes and professional help with people's mental health concerns’.^3^ The guidance warns that this positive effect – for instance, in ‘demystifying’ mental health problems – requires ‘proper attention to accurate portrayal’.

These statements suggest a professional common sense in which psychiatrists and psychologists engage in media work to supply a general audience with medical and scientific knowledge informed by relevant professional expertise. Benefits such as destigmatisation of mental illness ensue from improved public understanding. Media work should supply accurate information (which may mean conveying professional dissensus), and it should not do harm to individuals (for instance, by subjecting them to ill-informed and unwanted public diagnosis).

This professional common sense is outmoded. It suggests that media work by psychiatrists – and other professionals – appropriately simplifies expert knowledge for a public audience in deficit, while safeguarding them against non-expert errors and inaccuracies. Here, I draw upon theory and historical illustration to show how the professional common sense sketched above can be challenged by a richer account of the nature, opportunities and risks of media work by psychiatrists (and other mental health professionals). I offer my interrogations under four headings: motives for mass media work; the mass media as an industry; the messages of medium and form; and accruing and converting celebrity capital. I conclude with some recommendations and reflections.

## Motives for media work

As indicated above, professional guidance frames media work as fulfilment of a professional responsibility to public enlightenment and care. Media work is clearly not a duty for every professional, but it is nonetheless praiseworthy. Addressing a mass audience is going above and beyond the call of professional duty of beneficence toward individual patients and clients.

What remains unspoken are the realities of media work that complicate the simple picture of disinterested service to the well-being of the public (much as ethical complexities attend consultative work by psychiatrists on the behalf of organisations^4^). We might acknowledge, for instance, that inclination as well as duty motivates professionals who take on media work. Historiography has a useful role here in showing the rewards received, or hoped for, through media work. We can talk more frankly about the motives of actors in the recent past than we can about those of contemporaries who remain among the living.

Consider David Stafford-Clark, the ‘BBC's psychiatrist’ of the 1950s and 1960s and author of a highly successful 1952 Penguin introductory paperback, *Psychiatry To-day*. Media work was partly a vehicle for Stafford-Clark's literary vanity. He hoped that the commercial success of his laypersons’ guide would pave the way for literary renown as a celebrated national poet.^5^ The rising public status of the psychological disciplines encouraged this minor celebrity and committed Christian to use his soapbox in the mass media to pronounce on wider social and political issues, such as racism and sexuality. His moralising pronouncements eventually provoked a BBC editor to declare in exasperation, ‘who the devil is this dealer with sick people, to pontificate like this about the whole of human life?’.^6^

Stafford-Clark's career gives us a glimpse of the personal needs that media work can satisfy. In his case, they included literary ambition and a desire to offer spiritual and moral leadership in a secularising era. He also found his celebrity status gratifying. Whatever the mix of good and bad in his career, his motives are more complex than disinterested service to the enlightenment and well-being of the lay public.

## The mass media as an industry

The picture of the media psychiatrist as a disinterested educator misrepresents not merely their motives but also the reality of their work. The media psychiatrist is not an educator setting a syllabus and teaching a captive audience but rather an often freelance worker in an industry of mass production addressing a public who are consumers, not students. The product that the media psychiatrist offers is shaped by their economic position in many complex ways, whatever their intentions.

Consider the commissioning of a product, such as a book on a psychiatric or psychological topic for the mass market. Whatever the professional's views on what the public ought to know and be interested in, the contractual decision is critically informed by the publisher's judgement on the book's commercial prospects.

For instance, in Penguin Books’ post-war heyday, a non-fiction paperback in its Pelican imprint had to sell several thousand copies to be viable.^5^ Even this threshold depended on much higher sales elsewhere in its list. In commissioning psychiatrist J. A. C. Brown's *Techniques of Persuasion* (1963), on brainwashing,^7^ Penguin were consciously hoping to meet a lucrative market created by William Sargant's earlier bestseller *Battle for the Mind* (1957). Both books were the commercial result of a broader cultural fascination with the realities and possibilities of Cold-War brainwashing stimulated by historical events and amplified by journalistic, fictional and cinematic representations (in movies such as *The Manchurian Candidate* (1962) and *The Ipcress File* (1965)^8^).

Moreover, the model of the disinterested educator assumes that the media psychiatrist's commodities are consumed primarily or solely as information goods. This may be broadly plausible of the Highway Code or an Ordnance Survey map, but it seems a very partial view of the media psychiatrist's products. Goods may also be consumed as solidarity goods that facilitate a sense of belonging.^9^ Owning a copy of radical psychiatrist R. D. Laing's *The Politics of Experience* (1967) surely marked and facilitated a sense of countercultural membership, however deeply it was read.

The media industry brings its own idiosyncratic logic. An author or broadcaster may be recommissioned because they have proven to be a reliable supplier of products that are successful in terms of quantitative measures such as sales, viewing numbers, audience research and so forth. But consumers are also choosers: their interest may be dulled by overexposure to a personality or an issue. Overexposure befell David Stafford-Clark, curtailing his media career with the BBC, as surely as it did R. D. Laing, whose *Sonnets* seemed to bemoan the loss of his ‘funky charm’.^10^ Moreover, interest in mental health and illness waxes and wanes among the public, who have a finite capacity for multiple competing social problems in the public arena.^11^

## The messages of medium and form

The media in which psychiatrists work also bring implicit meanings. This is particularly obvious with broadcast media, which are particularly apt for the formation of a so-called para-social relationship between viewer and broadcaster. The viewer feels about their largely unilateral relationship with the broadcaster as they would about an everyday face-to-face relationship. Although sometimes denigrated as an ‘illusion’ of intimacy,^12^ the para-social meaning in the viewer–broadcaster relationship has analytic significance. The broadcaster has to talk with their audience, not at them, or down to them; they have to speak as if they were conducting an ordinary, unforced conversation with an equal.^13^

A psychiatrist in the broadcast media is, phenomenologically speaking, a guest in the viewer's (or listener's) private space. In the earlier days of television, this might mean a domestic room, though now media devices can accompany us almost anywhere. To patronise, offend, irritate or bore the viewer is to invite expulsion at the press of a button. Nor are such considerations limited to television. Even a radio psychiatrist must adapt their voice to a conversational style (as has been shown in D. W. Winnicott's career with BBC Radio^14,15^).

But the implicit sense of an egalitarian, freely chosen, personal relationship may belie the realities of everyday psychiatric practice. Should the viewer ever become a patient, they may find themselves in a bureaucratised and administered relationship, with no choice in their interlocutor, and in a significantly disempowered position. Psychiatrists encountered in medical care cannot be changed as readily as the channel or simply switched off and ignored. The broadcasting medium must necessarily misrepresent psychiatry in this respect.

Moreover, broadcasts come in definite forms that carry further meaning. The expectation of balanced news reporting may, for instance, create ‘false balance’ wherein a heterodox opinion becomes unduly authorised, as with the autism–vaccine controversy.^16^ Quasi-psychiatric formats have their own peculiar meanings. Consider the long-running BBC Radio programme *In the Psychiatrist's Chair* (1982–2001), in which psychiatrist Anthony Clare interviewed a wide variety of public figures.^17^ Contemporary academic analysis understands the programme's ‘therapy talk format’ as promoting a model of self-discovery in which a skilled intermediary helps to disclose a truer, inner self. This format became a model not only for the public to adopt but also an expectation upon well-known people that they should publicly perform scripts of transformative self-narration.^18^

The therapy talk format bears only a vague family resemblance to routine clinical psychiatric practice: no guest of *In the Psychiatrist's Chair* was diagnosed or treated for mental illness during these lengthy interviews, despite the programme's title and the occupation of the interviewer. An early reviewer in *The Times* concluded that there was ‘little more to this series than the prying impertinence of the popular journalist’.^19^ Psychiatry was there to lend its cultural and social capital to the para-social intimacy offered in a newer form of celebrity interview, one that had been pioneered in the late 1950s by journalist John Freeman in the BBC TV series *Face to Face*.^18^

## Accruing and converting celebrity capital

Media psychiatrists are to some extent celebrities. Their celebrity may initially be ‘achieved’ by virtue of their professional standing, but they will also gain ‘attributed’ celebrity by virtue of their sustained media representation.^20^ Celebrities from any background can use their celebrity as a kind of capital that allows them to ‘migrate’ into other fields.^21^

Stafford-Clark, as mentioned, used his fame to leverage literary endeavours, as did Laing. Clare was very active in Irish political networks and even stood (unsuccessfully) for a seat in the Irish Senate.^17^ All three tried on the mantle of the public intellectual,^22,23^ offering diagnoses of the age or, in Clare's case, of contemporary masculinity in crisis.^24^ To assume that these three figures were necessarily qualified for these activities would be naïve. They were using celebrity to accrue literary distinction, enter powerful networks, exercise cultural authority and wield political power.

The phenomenon continues in migration of other mental health professionals whose celebrity capital facilitates their entry into political networks and the processes of government – psychiatry is not alone in this regard. But professional models of media work seem indifferent to the contemporary significance of ‘celebritisation’, by which celebrity is increasingly detached from achievement, open to a wider range of entrants and used as a pathway into other fields of activity.^25^ At the very least, awareness of celebritisation might temper professional concern about unqualified media commentators on mental health and illness. The social media personality (or superfluous royal) who converts their celebrity capital into purported psychiatric authority is the shadow image of the psychiatrist who uses celebrity migration to diversify their own professional portfolio.

## Conclusion

The model persists of the media psychiatrist as a servant of the public good, expounding appropriately simplified information to a receptive public who thereby become enlightened citizens, better able to understand their own problems and needs and less likely to stigmatise persons with mental illness. For convenience, we might call this a literacy model, the public being construed as unlettered in the art of psychiatry and the media psychiatrist as a kindly schoolteacher.

This model is a partial truth. Whatever the rewards of disinterested service to the public, we might admit that the prospect of money, fame and a soapbox may be enticing. Who gets to fulfil the role of media psychiatrist, and what subjects they can speak about, is crucially shaped by the unpredictable needs of a complex media ecology and marketplace, to which the psychiatrist must adapt. The properties of the media and different forms also bring implicit meanings, such as the wider authorisation of therapeutic reflection as a path to authenticity or the promotion of para-social intimacy as a requirement of public figures. As a canny entrepreneur, the media psychiatrist may also convert the currency of celebrity into new and surprising forms of cultural, social and political capital.

The preceding argument sketches some of the ways in which professional self-understanding could (and I think should) be better informed. It is incomplete and provisional, but it may provoke further consideration in psychiatry and other mental health professions. I conclude by offering some recommendations and reflections.

Professional guidelines and advice should convey a more up-to-date and contextually richer understanding of the mass media marketplace, its nature, its opportunities and its risks. (Such guidelines should also be reviewed regularly and made easily available to the public as well as professionals.) The risks of media work are not easily captured within the framework of professional ethics, with their emphasis on principles guiding behaviour toward patients and clients. Ethical codes may even be a way of avoiding responsibility, thought and judgement.^26^ Historiography can help by offering narratively rich and ethically ambiguous case studies of media work.

The literacy model of media work by mental health professionals neglects the position of expertise by experience among patients and clients^27^ and therefore lags behind the more recent ‘contextual’ and ‘dialogic model of cooperation and negotiation between scientists and laypeople’.^28^ Professional boundary work^29^ to exclude non-expert perspectives may presume that illegitimate entrants to debate can be identified by their reliance on media mechanisms (such as the false balance problem noted earlier, or the processes of celebritisation). The non-expert or the ‘bad expert’^30^ may thereby be identified and denigrated as, say, an amateur or perhaps even a charlatan granted a platform by virtue of celebrity, motivated by a desire for money and renown, and exploiting followers – fans, in truth – enthralled by the illusion of para-social intimacy. Yet ‘good experts’ frequently use their celebrity to wander – or trespass – into other disciplines and domains, typically have their share of utilitarian motivations, and may similarly draw together a quasi-fan community from among their audiences. Media guidance for mental health professionals would do well to acknowledge the ubiquity of phenomena such as celebrity migration and para-sociality; the media psychiatrist cannot draw a magic circle in chalk to keep these forces at bay.

Media theory may also help psychiatry to clarify and implement the tricky ideal of ‘equitable dialogue’ with patient activists, service users, survivors and the wider public.^31^ Familiar elements of public engagement include not just a commitment to apparently non-hierarchical everyday conversation but also to formats such as the panel debate with a moderated question and answer session, familiar in the UK from radio and television programmes such as *Any Questions?* and *Question Time*. However, such event formats in public engagement with science position the facilitator role ‘as the ultimate locus of power’,^31^ belying their aspirations to a level playing field. We might speculate that common sense models of equitable dialogue for public engagement are shaped unwittingly – and to their detriment – by styles and formats derived from radio and TV broadcasting.

## About the author

**Gavin Miller** is Reader in Contemporary Literature and Medical Humanities in the School of Critical Studies at the University of Glasgow, Glasgow, UK.
